# Host‐Specificity and Network Structure of Tick Microbiota in Co‐Distributed Species From the Iberian Peninsula

**DOI:** 10.1002/ece3.71714

**Published:** 2025-07-04

**Authors:** Víctor Noguerales, José de la Fuente, Sandra Díaz‐Sánchez

**Affiliations:** ^1^ Departamento de Biología Animal, Edafología y Geología Universidad de La Laguna San Cristóbal de La Laguna Spain; ^2^ Instituto de Productos Naturales y Agrobiología (IPNA‐CSIC) San Cristóbal de La Laguna Spain; ^3^ Instituto de Investigación en Recursos Cinegéticos IREC‐CSIC‐UCLM‐JCCM, SaBio Ciudad Real Spain; ^4^ Department of Veterinary Pathobiology, Center for Veterinary Health Sciences Oklahoma State University Stillwater Oklahoma USA

**Keywords:** 16S rRNA amplicon metabarcoding, arthropod, microbial networks, microbiota, tick

## Abstract

Ticks are arthropods that have evolved a unique blood‐feeding lifestyle, making them intriguing subjects for exploring their adaptations to vertebrate hosts, their ability to transmit diseases, and their varied roles in the ecosystem. Despite the recent emphasis on the relevance of microbes in various aspects of tick biology (e.g., nutrition, metabolism, reproduction, survival, and competence), our understanding of microbial community variation across species and the underlying processes remains limited. Here, by integrating high‐throughput DNA sequencing with microbial ecological analyses and network association approaches, we investigate the community composition and structure of microbiota across natural populations of three co‐distributed tick species in the central Iberian Peninsula (Castilla‐La Mancha region). Our results revealed a complex and diverse microbiota, primarily composed of Proteobacteria, Bacteroidota, and Firmicutes. While all tick species exhibited a shared core microbiota, notable differences in microbial composition and network interactions suggest that each species may be influenced by ecological and evolutionary factors that shape their microbiota. These insights enhance our understanding of the complex relationships between ticks, their microbiota, and the surrounding environment, which can lead to improved strategies for managing vectors and controlling pathogens.

## Graphical abstract

We investigate the community composition and structure of microbiota across natural populations of three co‐distributed tick species in the central Iberian Peninsula. Our results revealed a complex and diverse microbiota, while all tick species exhibited a shared core microbiota. Notable differences in microbial composition and network interactions suggest that each species may be influenced by ecological and evolutionary factors that shape their microbiota.

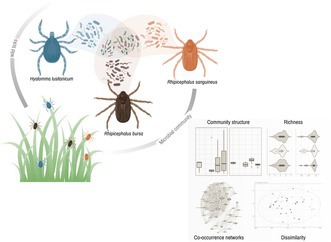

## 1 Introduction

Ticks (Acari: Ixodida) are parasitic arachnids that transitioned to a blood‐feeding lifestyle approximately 250 million years ago (Mans and Neitz 2004). This evolutionary path has turned ticks into excellent model organisms for examining their adaptations to different hosts, the development of vector abilities, and their ecological versatility (Jia et al. 2020; Mans 2021, 2023). In this context, it has been argued that tick–microbiota interactions can lead to paradigm shifts in relation to tick evolution (Mans 2023). The essential role of microbes in tick evolution has recently been emphasized in the literature (Duron et al. 2017; Díaz‐Sánchez et al. 2019; Mans 2023), and now there exists a growing consensus that microbes should be regarded as a key genetic component of the host (Henry et al. 2021). This well‐established idea highlights the importance of symbionts and endosymbionts in the evolutionary adaptability of ticks as vectors (Ahantarig et al. 2013; Vavre and Kremer 2014). These microbial alliances have been proven to influence tick nutrition (e.g., supplying vitamins and cofactors, enhancing blood‐feeding; Zhong et al. 2021; Kolo and Raghavan 2023), metabolism (e.g., facilitating nitrogen recycling, respiration, and osmoregulation; Gerhart et al. 2018; Olivieri et al. 2019), reproduction (e.g., boosting success and larval motility; Kagemann and Clay 2013; Zhang et al. 2017; Li et al. 2018), and survival (e.g., enhancing temperature tolerance; Neelakanta et al. 2010).

Recent research on tick–microbe interactions has advanced our understanding of tick biology and vector competence. These interactions have revealed dynamics at the microbiome‐pathogen interface, impacting pathogen transmission. For example, Narasimhan et al. (2014) demonstrated that disrupting the gut microbiota *of Ixodes scapularis* larvae allowed colonization by *Borrelia burgdorferi* , the causative agent of Lyme disease. *Borrelia*‐positive ticks from natural populations exhibited greater bacterial diversity compared to *Borrelia*‐negative ticks, highlighting the significance of microbial diversity in pathogen dynamics and vector competence (Chauhan et al. 2020; Brinkerhoff et al. 2020). The complex interactions between ticks, microbiota, and pathogens have also been observed with the obligate intracellular pathogen *Anaplasma phagocytophilum* , which modifies tick proteins to disrupt gut microbiota, thereby facilitating its transmission to the salivary glands (Abraham et al. 2017). Beyond facilitating pathogen transmission, tick–microbiota interactions may aid in adaptation to new environments and ultimately lead to host population differentiation (Gilbert et al. 2015; Mazel et al. 2018). For instance, Neelakanta et al. (2010) found that *A. phagocytophilum* infection enhanced tick tolerance to freezing by inducing the production of antifreeze glycoproteins, which may constitute an evolutionary opportunity under changing environments.

The composition of tick microbiota varies according to life stage, feeding status, host species, and local environmental conditions (Bonnet and Pollet 2021; Aivelo et al. 2022). However, the underlying processes and their relative roles in governing tick microbial community assembly remain unclear. Ticks may acquire their microbiota through maternal transmission and horizontal transfer from the environment, which could occur through the spiracles, mouth, and anal pore, or even during copulation (a paternal transmission route) or while feeding on host blood (Narasimhan and Fikrig 2015). The local environment may serve, therefore, as a significant microbial reservoir, fostering interactions that stimulate arthropod development and shape microbial community assembly (Guégan et al. 2018; Hannula et al. 2019; Girard et al. 2023). Although the genetic traits of tick hosts and their microbial interactions have not been thoroughly investigated, they are likely to play a crucial role in shaping the composition of tick microbiota (Frazenburg et al. 2013; Davenport 2016). Current research reveals only a few species‐specific connections in tick‐related microbiota, underscoring the significant relationships between ticks and specific microbes (Cabezas‐Cruz et al. 2018; Díaz‐Sánchez et al. 2019). By examining the species‐specific microbiota in wild tick populations, we can gain valuable insights into the factors that drive microbial dynamics. Such understanding is vital for comprehending vector competence, pathogen transmission, nutritional support, reproductive success, resilience to environmental stress, and immune regulation (Bonnet et al. 2017; Bonnet and Pollet 2021; Narasimhan et al. 2021).

We investigate the composition and structure of microbial communities across natural populations of three co‐distributed tick species ( *Hyalomma lusitanicum* , *Rhipicephalus sanguineus*, and *Rhipicephalus bursa* ) in the central Iberian Peninsula (Castilla‐La Mancha region). Specifically, we integrate high‐throughput DNA sequencing of tick microbiota with microbial ecological analyses and network association approaches to examine the patterns of microbiota richness, dissimilarity, and microbe–microbe associations within and among these tick species, which co‐occur at a microgeographic scale. Within this analytical context, we further assess the specificity of tick‐associated microbial communities and the extent to which they are shaped by a combination of abiotic factors (such as climate and vegetation) and biotic interactions, including competition among microbes and host‐specific dynamics. These findings might offer valuable insights into the microbiota‐driven mechanisms that influence tick ecology and may ultimately affect their role as disease vectors.

## 2 Materials and Methods

### 2.1 Tick Sampling and Taxonomic Identification

During the summer 2019/2020, unfed ticks were collected using the flagging‐dragging method (Mays et al. 2016) in five different locations within the region of Castilla‐La Mancha (Spain). This sampling yielded a total of 46 ticks from the species *H. lusitanicum* , *R. sanguineus* , and *R. bursa* , and three locations where two different tick species co‐occur (Table S1). Each collected tick was individually stored in new, sterilized 1.5 mL microcentrifuge tubes following a surface sterilization protocol in the laboratory involving successive washes: brief washes of 1 mL 3% hydrogen peroxide (1 min vortex), 70% ethanol twice (30 s each), and phosphate buffered saline solution (2 min) (Couper and Swei 2018). After surface sterilization, each tick was placed in a new, sterilized 1.5 mL microcentrifuge tube and frozen at −20°C before DNA extraction. All ticks collected were taxonomically identified at the genus and species levels using a stereomicroscope and following the taxonomic keys described by Manilla (1998).

### 2.2 DNA Extraction, Library Amplicon Preparation, and Sequencing

Genomic DNA from ticks was extracted individually utilizing the Qiagen DNeasy and Tissue kit (QIAGEN, Valencia, California, USA) following the manufacturer's instructions. The extraction process involved physically disrupting the ticks by bisecting the tissues with a scalpel (Ammazzalorso et al. 2015). DNA extracts were processed into individual genomic libraries for 16S rRNA gene amplicon sequencing using the V3 and V4 hypervariable regions, as detailed in Klindworth et al. (2013). Briefly, after PCR amplification, amplicon size (480 base pairs, bp) was verified using a Bioanalyzer DNA 1000 chip (Agilent, US) and then purified with AMPure XP beads (Beckman Coulter, Life Sciences, US). PCR products were used for a dual‐indexed library preparation following the Nextera XT DNA workflow (Illumina) and sequenced on a paired‐end 2 × 300‐bp lane of an Illumina MiSeq Platform. Genomic library preparation and Illumina sequencing were performed in the ‘Genomic Unit’ (Campus Moncloa, Universidad Complutense de Madrid, Spain).

### 2.3 Illumina Read Processing and Microbiota Analysis

Raw sequence analysis was performed using the DADA2 1.12 inference algorithm (Callahan et al. 2016) in R 4.0.1 (R Core Team 2021) on primer‐free reads to correct sequencing errors and create amplicon sequence variants (ASVs) for the tick microbial communities. The reads were quality‐filtered using the *FilterAndTrim()* function, which truncated the forward and reverse reads at 280 and 255 bp, respectively. Further, reads were removed if they had more than two errors in each forward and reverse read. Reads were merged after the inference of sequence variation with *learnErrors* and denoised functions. Chimeric sequences were eliminated with *RemoveBimeraDenovo()*, and bacterial taxonomy was assigned to ASVs using the classify‐learn naïve Bayes taxonomic classifier *assignTaxonomy* based on the SILVA database (release 138) (Yarza et al. 2014). The relative abundance of bacteria ASVs per individual was extracted from original outputs at the phylum, family, and genus levels for further analysis (Data S1). Then, technical filtering was applied to remove spurious ASVs, excluding those phyla represented by fewer than three ASVs and showing a relative abundance of < 0.05%. Likewise, ASVs observed fewer than five times in more than two tick samples were also discarded. The relative abundance of bacteria ASVs per tick individual was estimated at the phylum, family, and genus levels, with the five most abundant bacterial taxa featured at each level using the *tax_glom()* function in *phyloseq*. Core and shared microbiota at the genus level among tick species and within localities where different tick species were co‐distributed (locality 3, locality 4, and locality 5) were tallied using the *amp_venn()* function of the *ampvis2* R package (Andersen et al. 2018), considering the “*core*” genera shared by groups with an abundance ≥ 0.05% and present in 85% of the individuals.

### 2.4 Estimates of Alpha and Beta Diversity in Tick Microbiota

Prior to estimating alpha diversity, rarefaction analyses were performed using the random subsampling function, *rarefy_even_depth()*, to correct for samples with low and uneven sequencing depths (Hughes and Hellmann 2005; McMurdie and Holmes 2013). Then, ASV richness (alpha diversity) per individual for each tick species (*H. lusitanicum, R. sanguineus*, and *R. bursa* ) and genera (*Hyalomma* spp., *Rhipicephalus* spp.) was estimated using the Shannon‐Wiener index, Gini‐Simpson (1‐Simpson's original index), and InvSimpson indexes, as implemented in the *estimate_richness()* function in *phyloseq*. Patterns of ASV richness variation across tick species and genera were visualized using the *geom_violin()* function, implemented in the *ggplot2* R package (v 3.5.1) in R. Additionally, phylogenetic diversity metrics were calculated using the *phyloseq_phylo_div()* function as implemented in the *metagMisc* R package. Given that phylogenetic diversity measures are not statistically independent of species richness (Tucker and Cadotte 2013), the standardized effect size of phylogenetic diversity (ses.PD; Faith 1992) and the standardized effect size of mean pairwise distance within communities (Mean Pairwise Distance among all species in an assemblage, ses.MPD; Webb 2000; Webb et al. 2002) were computed. Phylogenetic diversity metrics were visualized using the *gg_violin()* function from the *ggpubr* R package (v 0.6.0). Finally, community dissimilarity in microbiota among tick species and genera was estimated by calculating Bray–Curtis distances and visualized through a principal coordinate analysis (PCoA) using *phyloseq*.

### 2.5 Statistical Analyses

To test for statistical differences in ASV richness (Shannon‐Wiener, Gini‐Simpson, and InvSimpson indexes) among tick species, non‐parametric Kruskal rank tests, as implemented in the *kruskal.test()* function from the *ape* R package (Paradis and Schliep 2019), were used. Statistical differences in ASV richness among tick genera were tested through the Wilcoxon rank sum test with continuity correction using the *wilcox.test()* function. At the level of tick species, post hoc analysis for multiple comparisons was performed using the *dunn.test()* function available in the *dunn.test* R package (Dinno 2017), adjusting the *p*‐values in accordance with the Benjamini‐Hochberg method.

Differences in microbiome composition among tick species and genera were tested for significance using permutational multivariate analyses of variance (PERMANOVA) as implemented in the *adonis2()* function from the *vegan* R package (Oksanen et al. 2022). Marginal effects of these variables (tick genera and tick species) were calculated after accounting for the effect of geography through including locality as a term within the PERMANOVA models. The assumption of homogeneous dispersion for PERMANOVA was tested using the *betadispr()* and *betadispr.permutest()* functions. At the level of tick species, post hoc analysis for multiple comparisons was performed using the *TukeyHSD()* function.

### 2.6 Tick Microbial Network Associations

Microbe–microbe association networks and changes in microbial connectivity between the two more abundant tick species ( *H. lusitanicum* and *R. sanguineus* ) were investigated through quantitative comparisons as implemented in NetCoMi (Peschel et al. 2021). These analyses were performed at two levels: (i) including all localities and individuals from the two tick species, and (ii) specifically for those localities where the two tick species are co‐distributed (localities 3 and 4). The tick species *R. bursa* was excluded from these analyses due to its reduced sample size. Briefly, a single microbial network was created by agglomerating ASVs into genera using SparCC (Sparse Correlations for Compositional data; Newman 2008). This method accounts for the compositional nature of the data through a centered log‐ratio transformation (Qannari et al. 2014; Zamkovaya et al. 2021). To reduce the computational nodes in the network analysis and enhance result interpretability, filter parameters were set to include only the 50 most frequent taxa and those with over 1000 total reads. Moreover, the “signed” distance metric was used where strong negative correlations increase dissimilarity. Network centrality was examined using the degree, betweenness, and closeness centrality metrics, which provide insights into the individual roles of taxa within the microbial community. These measures help identify hubs, or *keystone taxa*, as described by Peschel et al. (2021), which are associated with specific roles within the microbial community. Clusters of nodes, which are closely interconnected but have few connections outside their module, were identified using greedy modularity optimization (*cluster_fast_greedy*) using the *netAnalyze()* function. Hubs were characterized as nodes with an eigenvector centrality value exceeding the empirical 95% quantile of all eigenvector centralities in the network. Additionally, network properties were identified using the average path length, edge and vertex connectivity, modularity, and clustering coefficient metrics. The network was visualized by assigning colors to determined clusters and scaling node sizes according to the column sums of *clr‐transformed* taxa. Changes in the connectivity of microbial association networks between the species *H. lusitanicum* and *R. sanguineus* were examined using the *netCompare()* function, implementing a permutation test (setting *permTest* to TRUE) with *nPerm* set to 100 and *nPermRand* to 1000 for the Rand index testing against random cluster assignments for further reporting the 10 genera exhibiting the highest absolute group differences in degree, betweenness, and closeness centrality.

## 3 Results

### 3.1 Ticks Microbial Community Structure

**FIGURE 1:**
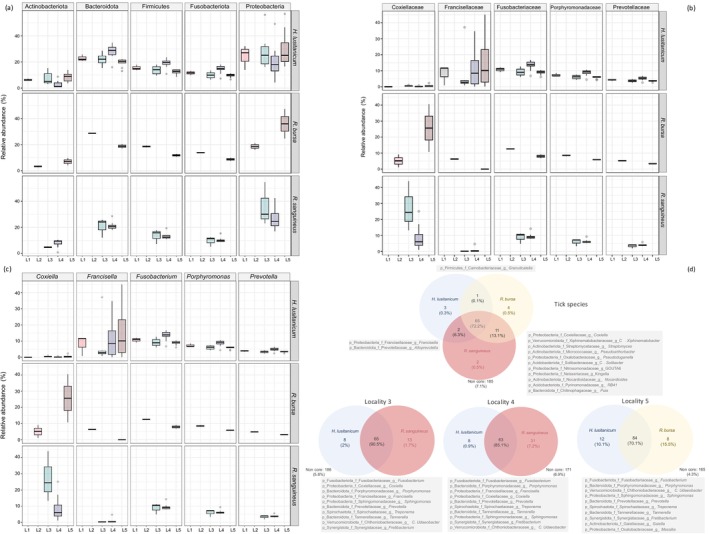
Boxplots showing the relative abundance of the five most abundant (a) phyla, (b) families, and (c) genera identified within the microbiota across tick species and localities. Venn diagrams (d) depict shared and unique genera of bacteria among the tick species *Hyalomma lusitanicum* and *Rhipicephalus* *sanguineus* across all localities and for only those where the two tick species co‐occur (localities 3, 4, and 5). Percentages in brackets represent the average abundance of these genera. Gray boxes indicate shared genera and the 10 most abundant shared genera in localities 3, 4, and 5.

Illumina sequencing provided a total of 17.06 M reads, with an average of 0.37 M reads per tick individual (Table S1). Quality and chimera filtering in DADA2 provided a total of 24,521 ASVs, which were assigned to 46 tick individuals, with further technical filtering yielding a data set comprising 1356 non‐spurious ASVs. Specifically, these ASVs clustered into a total of 24 phyla, 165 families, and 273 genera of bacterial taxa within the tick microbiota (see Data S1). Proteobacteria was the predominant phylum, accounting for 56% to 4.22% of all ASVs (Figure 1a), followed by Bacteroidota as the second most abundant phylum, with a range of 35% to 12% (Data S1: Dataset S1). The other three more abundant phyla were Firmicutes (23% to 7%), Fusobacteriota (23% to 7%), and Actinobacteriota (13% to 0.8%) (Figure 1a). At the genus level, the top five genera were *Coxiella*, *Francisella*, *Fusobacterium*, *Porphyromonas*, and *Prevotella* (Figure 1c) (Data S1: Dataset S3). *Coxiella* and *Francisella* exhibited similar relative abundances, ranging from 44% to 0%. Yet, *Coxiella* was more enriched in the tick genera *Rhipicephalus* ( *R. sanguineus* and *R. bursa* ), while *Francisella* was found to be more prevalent in ticks belonging to *H. lusitanicum* . The remaining three genera, *Fusobacterium* (16% to 4.7%), *Porphyromonas* (10% to 3.28%), and *Prevotella* (6.38% to 1.90%), were present in higher proportions, specifically within *H. lusitanicum* ticks (Figure 1c).

Overall analyses of core and shared microbiota at the bacteria genus level revealed that *R. bursa* and *R. sanguineus* shared a high proportion of their microbiota (89%; 76 out of 85 genera), as did *H. lusitanicum* with *R. sanguineus* (79%; 67 out of 84) and *R. bursa* (76%; 66 out of 86) (Figure 1d). These findings remained similar when analyses of core microbiota were restricted to localities where different tick species co‐occurred. At localities 3 and 4, *H. lusitanicum* and *R. sanguineus* shared 76% (65 out of 86 genera) and 61% (63 out of 102) of their microbiota, respectively. At locality 5, *H. lusitanicum* and *R. bursa* shared 80% of the genera (84 out of 104) in their core microbiota (Figure 1d). Remarkably, the most prevalent shared genera across all three tick species and localities included *Fusobacterium* (average relative abundance ranging from 13% to 11%) and *Porphyromonas* (also averaging 13% to 11%), followed by *Prevotella, Sphingomonas, Tannerella, Treponema*, and *C*. *udaeobacter*, which all had average relative abundances between approximately 5% and 3%. Other notable genera within the core microbiota of the three tick species were *Alistipes, Filifactor, Bacteroides, Streptococcus, Campylobacter, Lactobacillus*, and the *Lachnospiraceae* NK4A136 group, each with an average relative abundance of at least 1% (Data S2). We also noted the *Rickenellaceae* RC9 gut group, which had a lower representation in the core microbiota across the three tick species and locations, averaging around 0.17% (Data S2). Interestingly, genera such as *Coxiella* and *Francisella* were identified as part of the core microbiota only in localities 3 and 4, where *H. lusitanicum* and *R. sanguineus* co‐occurred, with average relative abundances of 10% to 5% and 7%, respectively (Data S2).

### 3.2 Richness and Dissimilarity in Tick Microbiota Community Composition

**FIGURE 2:**
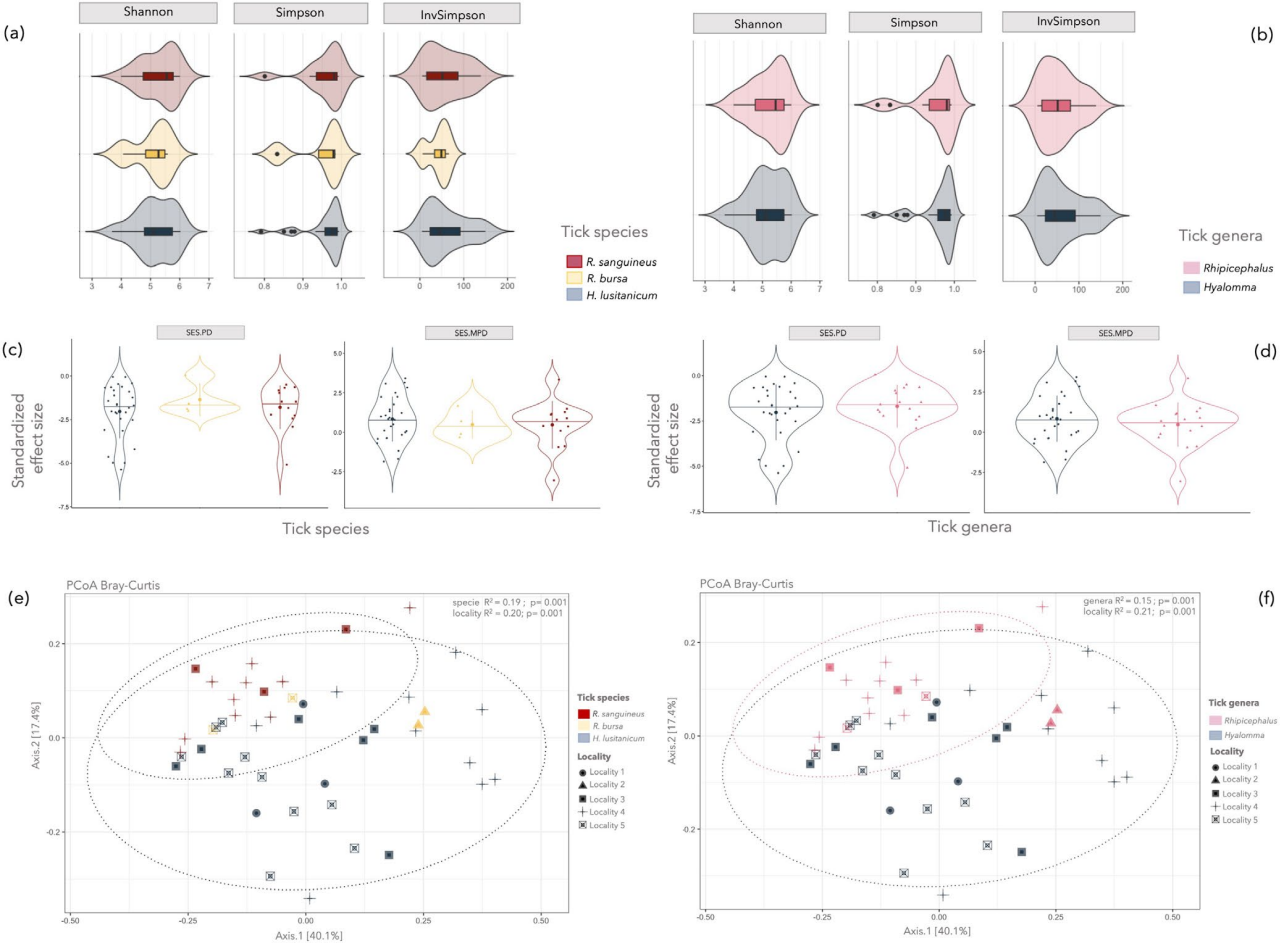
Upper panels represent violin plots illustrating the levels of richness (as estimated using the Shannon, Simpson, and InvSimpson indexes) and phylogenetic diversity (as estimated using the ses.PD and ses.MPD indexes) across tick species (panel a, c) and genera (panel b, d). The panel below illustrates the principal coordinate analysis (PCoA) of tick individuals based on microbiota dissimilarity. The percentage of explained variation (*R* ^*2*^) and the significance of tick species (e) and genera (f) as a grouping factor after controlling by locality based on PERMANOVA are reported on the top of the plot.

The average richness (alpha diversity) of ASVs per individual did not significantly differ among tick species (Figure 2a) for any of the richness indexes (Shannon: *X* ^2^ = 0.43, *p*‐value = 0.80; Simpson: *X* ^2^ = 0.22, *p*‐value = 0.89; InvSimpson *X* ^2^ = 0.22, *p*‐value = 0.89). Similar results were retrieved at the tick genera level (Figure 2b, Shannon: *W* = 234, *p*‐value = 0.70; Simpson: *W* = 258, *p*‐value = 0.80; InvSimpson; *W* = 258, *p*‐value = 0.80), indicating comparably highly diverse microbial communities at the two evolutionary scales (Table S1). The relatively high values of the Simpson index across the tick and genera species suggest a high probability of identifying diverse microbial taxa through random sampling of these microbial communities.

Estimation of phylogenetic diversity metrics revealed that tick microbial assemblages exhibited similar patterns at both tick species and genus levels. While ses.PD indicated a signature of phylogenetic clustering (negative values), the ses.MPD metric pointed toward a phylogenetic overdispersion of the community (positive values) (Figure 2c,d) (Table S1), with these inferences remaining similar at both the species and tick genus levels. However, as observed for alpha diversity indexes, phylogenetic structure did not significantly differ among tick species (Figure 2c) for either of the two estimators (ses.PD: *X* ^2^ = 0.48, *p*‐value = 0.74; ses.MPD: *X* ^2^ = 0.38, *p*‐value = 0.82). Similar results were retrieved at the tick genera level (Figure 2d; ses.PD: *W* = 226, *p*‐value = 0.64; ses.MPD, *W* = 272, *p*‐value = 0.56).

According to PCoA analyses based on Bray–Curtis distances, microbiotas of tick individuals largely grouped according to their respective tick species and genera (Figure 2e,f), although with a moderate degree of overlap. This analysis also revealed a relevant geographic correspondence in tick microbiota composition (Figure 2e,f). Accordingly, PERMANOVAs detected significant differences in microbiota composition among tick species (*R* ^2^ = 0.19) and genera (*R* ^2^ = 0.20), with the geographic origin of individuals significantly explaining as much as these two factors (locality: *R* ^2^ = 0.20–0.21). Finally, the Bray–Curtis dispersion within the different tick species and genera was not significantly different among groups (species: *F*‐value = 1.97, *p* = 0.10; genera: *F*‐value = 0.27, *p* = 0.60), indicating significant differences detected by PERMANOVA cannot be attributed to differing group centroids.

### 3.3 Tick Microbial Co‐Occurrence Networks

**FIGURE 3:**
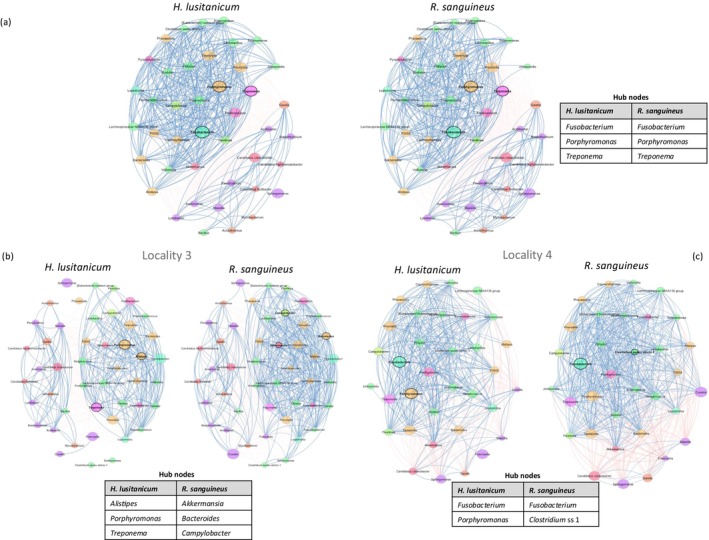
The microbial associations of *Hyalomma lusitanicum* and *Rhipicephalus sanguineus* observed across all localities (as illustrated in panel a), as well as in the two specific localities where the two tick species co‐occur (localities 3 and 4, respectively, as depicted in panels b and c). Blue edges indicate positive correlations, with red edges indicating negative correlations. Node colors indicate phylum, and sizes are clr‐transformed. Hubs (in bold) have eigenvector centrality above the 95th percentile.

The analysis of microbial networks revealed significant structural similarities between *H. lusitanicum* and *R. sanguineus* . Both tick species exhibited similar clustering patterns, with *Fusobacterium, Porphyromonas*, and *Treponema* as common hub nodes for both networks (Figure 3a; Table S2c). The properties of the species‐specific networks were largely similar, as indicated by their values in global metrics (frequency of clusters and detected hub nodes) and centrality measures for the 10 bacteria genera with the largest absolute differences (Table S2). No significant differences were found in global network metrics (Table S3) or centrality measures (Table S4), despite notable centrality variations in some bacteria taxa between tick species, particularly in *degree* and *closeness centrality*. The Jaccard index for *closeness* and *eigenvector centrality* was high (Jaccard = 0.883 for both) and statistically significant (*p* (*J* ≥ *j*) = 0.000), indicating a substantial overlap in the most centrally connected taxa, with *hub taxa* conserved across the two networks (Jaccard = 1.0; *p* (*J* ≥ *j*) = 0.037). However, *degree* and *betweenness centrality* showed no significant similarity, suggesting species‐specific variations in taxon connectivity and bridging (Table S5). The adjusted rand index (ARI) was very high (0.968, *p*‐value = 0), indicating nearly identical modular organization between the two microbial networks (Table S6).

In locality 3, the *H. lusitanicum* and *R. sanguineus* networks showed distinct clustering patterns and hub nodes (Figure 3b). Hub nodes for *H. lusitanicum* include *Alistipes, Porphyromonas*, and *Treponema*, while *Akkermansia, Bacteroides*, and *Campylobacter a*re key taxa in the *R. sanguineus* network (Table S7c). A comparison of centrality measures reveals that microbial taxa in *R. sanguineus* networks exhibit higher *degree* and *closeness centrality*, indicating a more connected and potentially efficient microbial structure than in *H. lusitanicum* . Notably, Candidatus *Xiphinematobacter* and *Pseudolabrys* show significant increases in closeness centrality (*p* < 0.001). In contrast, *betweenness centrality* was similar between species, suggesting comparable bridging roles within microbial networks. These results indicated a species‐specific structuring of the microbiota at the node level, with *R. sanguineus* possibly hosting more interactive and central microbial taxa (Table S9). The comparison of tick microbial networks showed no significant overlap in central taxa. All centrality metrics, including *eigenvector centrality*, showed low Jaccard indices (Jaccard < 0.33) and non‐significant *p*‐values, indicating that the core influential taxa differ substantially (Table S10). Notably, the complete absence of shared hub taxa is marginally significant (Jaccard = 0.000; *p* (*J* ≤ *j*) = 0.088), suggesting that the replacement of ecologically important hub nodes may not be as random as expected by chance. This result may suggest a potential host‐specific selection of key microbial members despite their co‐occurrence in the same community environment. Meanwhile, the ARI differs significantly from zero (ARI = 0.659; *p*‐value = 0.000), indicating that the overall modular structure is well conserved between the two tick species (Table S11). This result, along with previous observations, may suggest that although highly connected nodes differ between tick species—potentially reflecting host‐specific selection—the overarching organization of microbial interactions into functional or ecological modules demonstrates stability.

Networks of co‐occurring *H. lusitanicum* and *R. sanguineus* at locality 4 resulted in a distinct clustering pattern, similar to that from locality 3, except for one shared hub node: *Fusobacterium* (Figure 3c; Table S12c). Detailed global measures, as well as the 10 genera with the highest absolute group differences in *degree*, *betweenness*, and *closeness centrality*, are provided in Tables S13 and S14. Results from centrality measures indicated that most taxa, including *Peptostreptococcus, Francisella*, and *Alistipes*, showed significantly higher connectivity in *R. sanguineus* . However, no statistical evidence suggests that these differences are biologically significant. Similar to locality 3, the *R. sanguineus* microbial network appears to be more connected, as indicated by higher *degree centrality*; this could reflect a more structured or interactive microbiota in this tick species. However, differences in *betweenness centrality* and *closeness centrality* were less pronounced and not statistically significant (Table S14). Meanwhile, most connected, bridging nodes and *hub taxa* between the two microbial networks exhibited low Jaccard similarity (all below Jaccard ≤ 0.385) and were not significantly conserved across networks. In contrast, *eigenvector centrality* showed a moderate Jaccard similarity (Jaccard = 0.636), statistically significant (*p* (*J* ≥ *j*) = 0.038), indicating that highly connected taxa to other important nodes were largely conserved (Table S15). The ARI showed weak similarity (ARI = 0.220, *p* = 0.003) between the two microbial networks, suggesting that while the overall modular structures of both microbial networks are not well conserved in this locality, they still share more structure than expected by chance. These observations suggest that while the overall modular structures of both microbial networks are not well conserved in this locality, they still share more structure than expected by chance (Table S16).

## 4 Discussion

This study examined the microbial communities associated with natural populations of three tick species—*H. lusitanicum, R. sanguineus*, and *R. bursa* —collected in the central Iberian Peninsula (Castilla‐La Mancha, Spain). Previous research on the same region has documented these ticks, which often feed on Eurasian wild boar ( *Sus scrofa* ) and red deer ( *Cervus elaphus* ) (Fernández de Mera et al. 2013). Notably, *H. lusitanicum* is extensively distributed in the central Iberian Peninsula, contributing over 80% of the tick abundance in the observed community (Requena‐García et al. 2017; Valcárcel et al. 2014). This fact, together with their co‐occurrence at a microgeographic scale, may be indicative of similar ecological interactions and environmental factors. Accordingly, our results revealed that at the phylum level, Proteobacteria were predominant in all three tick species, consistent with earlier studies conducted both in the Iberian Peninsula and worldwide (Narasimhan and Fikrig 2015; Portillo et al. 2019; Díaz‐Sánchez et al. 2021). Other phyla, such as Bacteroidota, Firmicutes, Actinobacteriota, and Fusobacteriota, were also significant, highlighting the diversity of tick‐associated microbiota. Noteworthy findings at the genus level include the predominance of *Coxiella* and *Francisella*. These genera exhibit a range of behaviors that encompass critical endosymbiotic functions essential for the survival of ticks, as well as pathogens implicated in diseases such as Q fever ( *Coxiella burnetii* ) and tularemia ( *Francisella tularensis* ) (Gerhart et al. 2016; Andreotti et al. 2011; Ben‐Yosef et al. 2020; Duron et al. 2015, 2017). The presence of the genus *Francisella* in *H. lusitanicum* and *Coxiella* in *R. sanguineus* and *R. bursa* is consistent with earlier findings (Toledo et al. 2009; Díaz‐Sánchez et al. 2021; Herrera et al. 2023), suggesting that evolutionary factors may drive these species‐specific microbial associations. Additionally, we observed a significant community of shared genera among tick species, indicating a likely well‐conserved core microbiota (Figure 1d), as previously reported by Xu et al. (2024). Prior research has shown that ticks in close geographical proximity tend to share microbes (Portillo et al. 2019; Van Treuren et al. 2015), a pattern that has often been explained as the result of co‐feeding behavior, through which two or more ticks share microbes by feeding on the same host (Aivelo et al. 2022). Among the shared genera, *Fusobacterium, Porphyromonas*, and *Prevotella* were the most prevalent. *Fusobacterium* is a widespread bacterium found in soil, manure, the human gastrointestinal tract, and on the skin and hooves of domestic animals. It has also been identified as a commensal in tick microbiota (Clark et al. 1985; Tan et al. 1996; Fountain‐Jones et al. 2023). *Porphyromonas* has been detected in soil and within the intestinal microbiota of arthropods (Acuña‐Amador and Barloy‐Hubler 2020). Numerous microorganisms have been found to be linked to the soil in ecosystems where these arthropods thrive, emphasizing the role of environmental microbes in shaping tick microbial communities (Portillo et al. 2019; Narasimhan et al. 2021). *Prevotella* was observed in *H. lusitanicum, I. persulcatus* (Zhang et al. 2014; Díaz‐Sánchez et al. 2021), as well as in kissing bugs (Hemiptera: Triatominae) (Montoya‐Porras et al. 2018). The potential functional roles of these genera, including *Prevotella*'s contribution to blood digestion and *Fusobacterium*'s role in environmental nutrient cycling, merit further exploration to clarify their impact on tick biology and their competence as secondary symbionts (Folk and Leung 2002). A general understanding of tick bacteria microbiota and how OTU/ASV abundance affects functionality is still limited. While abundant OTUs are often viewed as functionally significant, it is crucial to recognize that rare OTUs/ASVs can also have a substantial effect on interactions between the host and microbes (Lozupone et al. 2012; Aivelo et al. 2022).

Analysis of phylogenetic diversity revealed that the microbial community structure of the three tick species follows both a clustering pattern and an overdispersion pattern, suggesting that deterministic processes such as competitive exclusion and habitat filtering influence the microbiota associated with these three tick species. This apparent conflict can be reconciled when considering processes at different evolutionary scales, which may lead to an overdispersion of clusters, generating both basal overdispersion and terminal clustering (Mazel et al. 2016; Goberna, Navarro, et al. 2014; Goberna, García, and Verdú 2014). These results align with the patterns of “basal” overdispersion, where the co‐occurrence of distantly related bacteria reflects host‐specific processes structuring the bacterial assemblages, potentially favoring certain bacterial lineages (Bevins and Salzman 2011; Hooper et al. 2012; Mazel et al. 2016). Consequently, microbial competition‐driven coexistence may produce phylogenetic overdispersion patterns, suggesting that distantly related species with minimal niche overlap and competition are more likely to coexist. In contrast, phylogenetic clustering is believed to signify the coexistence of closely related species sharing a similar environmental niche (Mazel et al. 2016). A prior study by Díaz‐Sánchez et al. (2019) found overdispersion patterns using ses.MDP metrics in *Ixodes affinis, I. scapularis, I. ricinus*, and *I. ovatus* microbial communities, while *Ixodes persulcatus, I. pavlovskyi*, and *I. ventalloi* exhibited phylogenetic clustering. Although these findings are significant, further studies should investigate the connection between these patterns and the ecological processes, as well as whether they relate to a continuum of abiotic and biotic factors influencing the assembly of the tick microbial community. Currently, we can only speculate about the processes potentially involved in tick microbial assembly. For instance, the hematophagous behavior of ticks may necessitate a complex digestive mechanism that requires various biochemical compounds for the degradation process (Mans and Neitz 2004). This may lead to the emergence of distinct bacterial clades that possess unique enzymatic profiles within the tick microbiota, likely resulting in overdispersion patterns characterized by the co‐occurrence of distantly related lineages. Within these clades, host‐specific processes like mucus barriers, oxygen levels, and the tick immune system may favor certain lineages across a broad phylogenetic scale. Indeed, feeding from a single host individual at each life stage can enhance opportunities for deterministic processes in microbial assembly (Aivelo et al. 2022). The beta‐diversity metrics analysis revealed that host‐specific and locality‐specific factors have a significant influence on tick microbiota composition. This finding underscores the importance of conducting longitudinal studies to monitor microbial dynamics across diverse tick species and environmental gradients. Notably, the visualization of beta diversity illustrates the presence of overlapping microbial communities among different tick species, particularly between *H. lusitanicum* and *R. bursa* . This overlap may suggest shared environmental conditions, as ticks residing in similar geographic areas might harbor a similar microbiota (Portillo et al. 2019; Xu et al. 2024). A similar pattern was observed by Herrera et al. (2023), who reported a corresponding pattern of beta diversity in ticks from Castilla‐Leon (northwestern Spain), where *R. bursa* and *R. sanguineus* clustered similarly with *Hyalomma marginatum*.

Microbial co‐occurrence networks revealed key taxa, including *Fusobacterium*, *Porphyromonas*, and *Treponema*, which were consistently identified across various tick species and locations in this study. The significance of *Fusobacterium* as a central taxon has been previously noted as an ecological indicator for pathogens like *Anaplasma* (Fountain‐Jones et al. 2023). In our results, we observed variations in microbial networks that pointed out that tick host and locality interact to shape microbial network structure, with some environments promoting convergence and others favoring host‐specific configurations at both node and module levels. Further research is needed to gain more understanding of the biological implications of these findings and how pathogens and symbionts interact with tick species, with environmental microbes influencing these dynamics. A study by Xu et al. (2024) revealed that environmental microorganisms are pivotal in microbial co‐occurrence networks, displaying fluctuating patterns among various groups. This aligns with our findings, suggesting that soil microbes could impact the composition of tick microbiota (Narasimhan and Fikrig 2015; Kwan et al. 2017). Literature indicates that *Porphyromonas* has been detected in the intestinal microbiota of arthropods, including ectoparasites and insects, and has been linked to human‐altered soils (agricultural land and crops) (Acuña‐Amador and Barloy‐Hubler 2020). *Treponema* species, which are obligate parasites found in a wide range of animal hosts, have some basal species that might be free‐living (Norris et al. 2006). *Treponema* can be both pathogenic and non‐pathogenic, sometimes playing a symbiotic role in hosts; for instance, in termite guts, they contribute to H_2_‐CO_2_ acetogenesis and nitrogen fixation, supplying carbon and energy in return for nitrogen (Graber et al. 2004; Gogarten et al. 2016). In *H. lusitanicum* , we noted a slightly lower clustering coefficient, alongside higher modularity and positive edge percentage compared to *R. sanguineus* , although these differences were not statistically significant across all tested hypotheses. In tick microbial networks, positive associations are significantly more prevalent than negative ones (Aivelo et al. 2022; Lejal et al. 2021; Fountain‐Jones et al. 2023). Fountain‐Jones et al. (2023) emphasized the vital importance of beneficial microbial relationships in influencing the microbiota of *I. scapularis* . The researchers discovered that positive interactions are common, implying a facilitation process and a trend of simultaneous colonization. Consequently, it is plausible that ticks may acquire multiple microbial groups at once. A positive edge percentage may suggest enhanced connectivity among nodes, which could increase the risk of transmitting an external stressor throughout the ecosystem, potentially harming the host (Kajihara and Hynson 2024). These network analyses elucidate the intricate relationships between environmental microbes and tick microbiota, shedding light on possible pathways for pathogen acquisition and transmission. Quantifying associations in natural settings and their relative significance against other ecological drivers presents a methodological challenge. Accounting for spatial and temporal patterns is essential, as species may co‐occur less often than expected by chance due to spatial segregation or limitations in dispersal rather than microbial competition (Fountain‐Jones et al. 2023). The analysis revealed discrepancies in the microbial networks of co‐occurring tick species. Specifically, the microbial taxa associated with *R. sanguineus* exhibit a higher degree of centrality and closeness centrality, suggesting a more interconnected and potentially efficient microbial structure compared to *H. lusitanicum* . This observation is supported by previous studies, which show that *R. sanguineus* demonstrates greater stability and robustness compared to the *R. turanicus* network (Maitre et al. 2024). Co‐occurring tick species may exhibit differences in pathogen competence due to variations in host preferences, unique physiological traits, and pathogen specificity (McCoy et al. 2013; de la Fuente et al. 2017). Moreover, biotic factors like humidity, temperature, vegetation, and environmental microbiota play pivotal roles in these tick–host–pathogen dynamics (Couret et al. 2022). Even more compelling is the insight that the complexity of their microbial community may provide advantages, allowing them to adapt to environmental stressors and resist pathogen colonization (Mota et al. 2023; Pavanelo et al. 2023).

## 5 Conclusions

This study presents a scenario in which wild ticks harbor a rich and diverse bacterial microbiota, indicating a stable core microbiota. We observed interesting divergences in the microbial network of co‐occurring tick species, which may be the result of species‐specific dynamics or environmental influences.

Despite the observed differences, the overarching organization of tick–microbial interactions remains stable. The preservation of community structure alongside the turnover of key taxa suggests a scenario of functional convergence or redundancy, wherein various microbial entities maintain analogous ecological roles. The findings of this study pave the way for further research on how functional microbiota shape tick biology, particularly regarding shared and species‐specific microbial assemblages. Future efforts should focus on (i) longitudinal studies to assess the temporal stability of core tick microbiota; (ii) experimental research on essential genera like *Francisella* and *Coxiella*, but also on complex interactions between environmental microbes and tick microbiota, highlighting potential pathways for pathogen acquisition and transmission; (iii) integrative methods combining ecological, genetic, and environmental data to reveal factors influencing microbial community assembly. This work highlights the significance of microbiota in tick vector competence, paving the way for microbiota‐focused interventions in controlling vector‐borne diseases.

## Author Contributions

**Víctor Noguerales:** conceptualization (equal), data curation (equal), formal analysis (equal), investigation (equal), methodology (equal), supervision (equal), validation (equal), visualization (equal), writing – original draft (equal), writing – review and editing (equal). **José de la Fuente:** writing – review and editing (supporting). **Sandra Díaz‐Sánchez:** conceptualization (equal), data curation (equal), formal analysis (equal), funding acquisition (lead), investigation (equal), methodology (equal), project administration (lead), resources (lead), supervision (equal), validation (equal), visualization (equal), writing – original draft (equal), writing – review and editing (equal).

## Conflicts of Interest

The authors declare no conflicts of interest.

## Supporting information


**Data S1** Percentage of relative abundance of bacteria taxonomic assignations at phylum, family and genus level per host sample generated with DADA2 and grouped per tick species and locality.

**Data S2** Shared core microbiota genera based on tick species and their localities (species co‐occurring together).

**Table S1** Description of samples, collection sites and Alpha diversity estimations.
**Table S2** Properties of the networks shown in Figure 3a: (a) Frequency of clusters in the tick species *H. lusitanicum* network. (b) Frequency of clusters in the tick species *R. sanguineus* network. (c) Detected hub nodes in both groups (d–f).
**Table S3** Comparison of network properties considering all localities where *H. lusitanicum* and *R. sanguineus* were present (Figure 3a).
**Table S4** Results from analyses testing for differences between centrality measures of the species‐specific networks shown in Figure 3a.
**Table S5** The Jaccard index quantifies the similarity of the sets of most central nodes and the sets of hub taxa between the microbial networks of tick species *H. lusitanicum* and *R. sanguineus* . Jaccard’s index is 0 if the sets of top‐ranking taxa for each centrality measure are completely different and 1 for exactly equal sets.
**Table S6** The adjusted rand index (ARI) quantifies the similarity between the structures in the *H. lusitanicum* and *R. sanguineus* networks, with values close to 1 indicating high agreement or identical clustering and 0 to the expected value for two random clusterings.
**Table S7** Properties of the networks shown in Figure 3b: (a) Frequency table of clusters in the tick species *H. lusitanicum* network in locality 3. (b) Frequency table of clusters in the tick species *R. sanguineus* network in locality 3. (c) Detected hub nodes in both groups. (d–f) Centrality values of the bacteria genera with the highest centrality in decreasing order.
**Table S8** Comparison of network properties considering the tick species *H. lusitanicum* and *R. sanguineus* collected from locality 3 (Figure 3b) for group differences.
**Table S9** Results from analyses testing for differences between centrality measures of the species‐specific networks shown in Figure 3b.
**Table S10** The Jaccard index quantifies the similarity of the sets of most central nodes and the sets of hub taxa between the microbial networks of tick species *H. lusitanicum* vs. *R. sanguineus* collected in locality 3.
**Table S11** The adjusted rand index (ARI) quantifies the similarity between clustering structures in the *H. lusitanicum* and *R. sanguineus* microbial networks collected from locality 3, with values close to 1 indicating high agreement or identical clustering and 0 to the expected value for two random clusterings.
**Table S12** Properties of the networks shown in Figure 3c: (a) Frequency table of clusters in the tick species *H. lusitanicum* network in locality 4. (b) Frequency table of clusters in the tick species *R. sanguineus* network in locality 4. (c) Detected hub nodes in both groups. (d–f) Centrality values of the bacteria genera with the highest centrality in decreasing order.
**Table S13** Comparison of network properties considering tick species *H. lusitanicum* and *R. sanguineus* collected from locality 4 (Figure 3c) for group differences.
**Table S14** Results from analyses testing for differences between centrality measures of the species‐specific networks shown in Figure 3c.
**Table S15** The Jaccard index quantifies the similarity of the sets of most central nodes and the sets of hub taxa between the microbial networks of tick species *H. lusitanicum* vs. *R. sanguineus* collected in locality 4.
**Table S16** The adjusted rand index (ARI) quantifies the similarity between clustering structures in the *H. lusitanicum* and *R. sanguineus* microbial networks collected from locality 4, with values close to 1 indicating high agreement or identical clustering and 0 to the expected value for two random clusterings.

## Data Availability

Raw Illumina reads were deposited at the NCBI Sequence Read Archive (SRA) under BioProject PRJNA1270924. Input files for all analyses are available for download from the Zenodo Digital Repository using the DOI 10.5281/zenodo.15582597. All Supporting Information tables cited in the main text have been uploaded as Supporting Information.
