# Supplementary material for: Host‐Specificity and Network Structure of Tick Microbiota in Co‐Distributed Species From the Iberian Peninsula
Source: Ecol Evol. 2025 Jul 4;15(7):e71714. doi: 10.1002/ece3.71714 (PMC12231218; doi:10.1002/ece3.71714)
Supplement: Supplementary file 3 — Table S1 Description of samples, collection sites and Alpha diversity estimations. Table S2 Properties of the networks shown in Figure 3a: (a) Frequency of clusters in the tick species H. lusitanicum network. (b) Frequency of clusters in the tick species R. sanguineus network. (c) Detected hub nodes in both groups (d–f). Table S3 Comparison of network properties considering all localities where H. lusitanicum and R. sanguineus were present (Figure 3a). Table S4 Results from analyses testing for differences between centrality measures of the species‐specific networks shown in Figure 3a. Table S5 The Jaccard index quantifies the similarity of the sets of most central nodes and the sets of hub taxa between the microbial networks of tick species H. lusitanicum and R. sanguineus . Jaccard’s index is 0 if the sets of top‐ranking taxa for each centrality measure are completely different and 1 for exactly equal sets. Table S6 The adjusted rand index (ARI) quantifies the similarity between the structures in the H. lusitanicum and R. sanguineus networks, with values close to 1 indicating high agreement or identical clustering and 0 to the expected value for two random clusterings. Table S7 Properties of the networks shown in Figure 3b: (a) Frequency table of clusters in the tick species H. lusitanicum network in locality 3. (b) Frequency table of clusters in the tick species R. sanguineus network in locality 3. (c) Detected hub nodes in both groups. (d–f) Centrality values of the bacteria genera with the highest centrality in decreasing order. Table S8 Comparison of network properties considering the tick species H. lusitanicum and R. sanguineus collected from locality 3 (Figure 3b) for group differences. Table S9 Results from analyses testing for differences between centrality measures of the species‐specific networks shown in Figure 3b. Table S10 The Jaccard index quantifies the similarity of the sets of most central nodes and the sets of hub taxa between the microbial netw [file ECE3-15-e71714-s002.zip › ece371714-sup-0004-TableS2-S16@SuppInfo 3.docx]

**Additional results for whole network properties of *H. lusitanicum* and *R. sanguineus*, considering all localities where these tick species were present.**

**Table S2.** Properties of the networks shown in **Figure 3a**: a) Frequency of clusters in the tick species *H. lusitanicum* network. b) Frequency of clusters in the tick species *R. sanguineus* network. c) Detected hub nodes in both groups. d-f) Centrality values of the bacteria genera with the highest centrality in decreasing order. The tables contain the five bacteria genera with the highest centrality in *H. lusitanicum* and *R. sanguineus*. A bacteria genus can occur twice in the same table following Peschel et al. (2021).

| **a) Cluster at *H. lusitanicum*** | |
| --- | --- |
| **Name** | **Frequency** |
| 0 | 3 |
| 1 | 28 |
| 2 | 13 |

| **b) Cluster at *R. sanguineus*** | |
| --- | --- |
| **Name** | **Frequency** |
| 0 | 2 |
| 1 | 28 |
| 2 | 14 |

| **c) Hub nodes. Based on empirical quantiles of centralities** | |
| --- | --- |
| ***H. lusitanicum*** | ***R. sanguineus*** |
| *Fusobacterium* | *Fusobacterium* |
| *Porphyromonas* | *Porphyromonas* |
| *Treponema* | *Treponema* |

| **d) Degree** | | |
| --- | --- | --- |
| **Genus** | | |
| **Highest values on the *H. lusitanicum* group:** | ***H. lusitanicum*** | ***R. sanguineus*** |
| *Fretibacterium* | 0.813 | 0.906 |
| *Campylobacter* | 0.813 | 0.906 |
| *Filifactor* | 0.813 | 0.860 |
| *Dialister* | 0.813 | 0.883 |
| *Streptococcus* | 0.790 | 0.883 |
| **Highest values on the *R. sanguineus* group:** | ***H. lusitanicum*** | ***R. sanguineus*** |
| *Fretibacterium* | 0.813 | 0.906 |
| C. *Udaeobacter* | 0.720 | 0.906 |
| *Campylobacter* | 0.813 | 0.906 |
| *Gaiella* | 0.790 | 0.906 |
| *Massilia* | 0.720 | 0.906 |

| **e) Betweennes centrality** | | |
| --- | --- | --- |
| **Genus** | | |
| **Highest values on the *H. lusitanicum* group:** | ***H. lusitanicum*** | ***R. sanguineus*** |
| *Fretibacterium* | 0.065 | 0.040 |
| *Gaiella* | 0.053 | 0.008 |
| *Arenimonas* | 0.038 | 0.009 |
| *Massilia* | 0.032 | 0.020 |
| *Acidibacter* | 0.026 | 0.000 |
| **Highest values on the *R. sanguineus* group:** | ***H. lusitanicum*** | ***R. sanguineus*** |
| *Fretibacterium* | 0.065 | 0.040 |
| C. *Solibacter* | 0.000 | 0.032 |
| *Streptococcus* | 0.000 | 0.026 |
| *Massilia* | 0.032 | 0.020 |
| C. *Udaeobacter* | 0.015 | 0.013 |

| **f) Eigenvector centrality** | | |
| --- | --- | --- |
| **Genus** | | |
| **Highest values on the *H. lusitanicum* group:** | ***H. lusitanicum*** | ***R. sanguineus*** |
| *Fusobacterium* | 1.000 | 1.000 |
| *Porphyromonas* | 0.990 | 0.998 |
| *Treponema* | 0.990 | 0.992 |
| *Filifactor* | 0.988 | 0.962 |
| *Tannerella* | 0.986 | 0.991 |
| **Highest values on the *R. sanguineus* group:** | ***H. lusitanicum*** | ***R. sanguineus*** |
| *Fusobacterium* | 1.000 | 1.000 |
| *Porphyromonas* | 0.990 | 0.998 |
| *Treponema* | 0.990 | 0.992 |
| *Tannerella* | 0.986 | 0.991 |
| *Streptococcus* | 0.948 | 0.990 |

**Table S3.** Comparison of network properties considering all localities where *H. lusitanicum* and *R. sanguineus* were present. (**Figure 3a)**. The table shows the results from testing global network metrics, their absolute difference, and their respective p-values. A p-value <0.05 indicates species-specific network measures are statistically different. Global network properties are defined for the whole network and offer information on the overall network structure (Peschel et al., 2021).

| **Properties** | ***H. lusitanicum*** | ***R. sanguineus*** | **Absolute difference** | **p-value** |
| --- | --- | --- | --- | --- |
| **Number of components** | 4.000 | 3.000 | 1.000 | 0.712 |
| **Clustering coefficient** | 0.883 | 0.899 | 0.016 | 0.812 |
| **Modularity** | 0.054 | 0.027 | 0.027 | 0.396 |
| **Positive edge percentage** | 68.850 | 58.126 | 10.724 | 0.069 |
| **Edge density** | 0.597 | 0.722 | 0.125 | 0.366 |
| **Natural connectivity** | 0.424 | 0.402 | 0.021 | 0.792 |

**Table S4.** Results from analyses testing for differences between centrality measures of the species-specific networks shown in **Figure 3a**. These results contain 10 subjects with the highest absolute group difference. All measures are normalized to [0,1]. Table shows the computed measures for *H. lusitanicum* and *R. sanguineus*, their absolute difference, and their respective p-values (Peschel et al., 2021). A p-value <0.05 indicates species-specific network measures are statistically different. The p-values are adjusted for multiple testing using the adaptive Benjamini-Hochberg method (Benjamini and Hochberg, 2000), according to Langaas et al. (2005).

|  | ***H. lusitanicum*** | ***R. sanguineus*** | **Absolute difference** | **p-value** |
| --- | --- | --- | --- | --- |
| **Degree (normalized)** | | | | |
| *Mycobacterium* | 0.047 | 0.791 | 0.744 | 0.625 |
| *Bacillus* | 0.279 | 0.907 | 0.628 | 0.607 |
| *Bradyrhizobium* | 0.093 | 0.698 | 0.605 | 0.885 |
| *Sphingomonas* | 0.000 | 0.558 | 0.558 | 0.485 |
| *Lachnospiraceae NK4A136 group* | 0.767 | 0.279 | 0.488 | 0.625 |
| *Candidatus Solibacter* | 0.209 | 0.698 | 0.488 | 0.520 |
| *Johnsonella* | 0.349 | 0.047 | 0.302 | 0.885 |
| *Fusobacterium* | 0.628 | 0.884 | 0.256 | 0.485 |
| *Porphyromonas* | 0.628 | 0.884 | 0.256 | 0.485 |
| *Treponema* | 0.628 | 0.884 | 0.256 | 0.485 |
| **Betweenness centrality (normalized)** | | | | |
| *Gaiella* | 0.054 | 0.009 | 0.045 | 0.925 |
| Candidatus *Solibacter* | 0.000 | 0.033 | 0.033 | 0.925 |
| *Arenimonas* | 0.038 | 0.010 | 0.029 | 0.925 |
| *Acidibacter* | 0.027 | 0.000 | 0.027 | 0.925 |
| *Streptococcus* | 0.000 | 0.027 | 0.027 | 0.925 |
| *Fretibacterium* | 0.065 | 0.040 | 0.025 | 0.925 |
| *Fusobacterium* | 0.022 | 0.005 | 0.017 | 1.000 |
| *Dialister* | 0.017 | 0.000 | 0.017 | 0.925 |
| *Filifactor* | 0.013 | 0.000 | 0.013 | 0.925 |
| *Massilia* | 0.032 | 0.021 | 0.011 | 0.925 |
| **Closeness centrality (normalized)** | | | | |
| Candidatus *Xiphinematbacter* | 0.742 | 2.029 | 1.287 | 0.292 |
| Candidatus *Solibacter* | 0.928 | 2.167 | 1.238 | 0.292 |
| *Sphingomonas* | 0.000 | 1.086 | 1.086 | 0.292 |
| *Pseudolabrys* | 1.107 | 2.085 | 0.978 | 0.292 |
| *Acidothermus* | 1.087 | 1.941 | 0.854 | 0.292 |
| *Gaiella* | 1.261 | 2.042 | 0.782 | 0.292 |
| *Bacillus* | 0.894 | 1.674 | 0.780 | 0.292 |
| *Massilia* | 1.324 | 2.102 | 0.778 | 0.292 |
| *Mycobacterium* | 0.736 | 1.395 | 0.659 | 0.600 |
| *Bradyrhizobium* | 0.795 | 1.403 | 0.608 | 0.600 |

**Table S5**. The Jaccard index quantifies the similarity of the sets of most central nodes and the sets of hub taxa between the microbial networks of tick species *H. lusitanicum* and *R. sanguineus*. Jaccard’s index is 0 if the sets of top-ranking taxa for each centrality measure are completely different and 1 for exactly equal sets. P(J ≥ j) indicates the probability of obtaining Jaccard’s index equal to or greater than the observed value under the null hypothesis of no difference between networks ((P(J ≤ j) is deﬁned analogously) (Peschel et al., 2021).

|  | **Jaccard index** | **P(J ≤ Jacc)** | **P(J ≥ Jacc)** |
| --- | --- | --- | --- |
| **Degree** | 0.250 | 0.468 | 0.804 |
| **Betweenness centrality** | 0.330 | 0.618 | 0.595 |
| **Closeness centrality** | 0.833 | 0.999 | 0.000 |
| **Eigenvector centrality** | 0.833 | 0.999 | 0.000 |
| **Hub taxa** | 1.000 | 1.000 | 0.037 |

**Table S6.** The Adjusted Rand index (ARI) quantifies the similarity between the structures in the *H. lusitanicum* and *R. sanguineus* networks, with values close to 1 indicating high agreement or identical clustering and 0 to the expected value for two random clusterings. Consequently, positive values imply that two clusters are more similar, and negative values are less similar than expected at random. The p-value was calculated via permutation (n=1000) to test the significance of the observed clustering agreement (Peschel et al., 2021).

| **Adjusted Rand index** | |
| --- | --- |
|  | **Whole network** |
| **ARI** | 0.968 |
| **p-value** | 0.000 |

**Additional results for whole network properties of *H. lusitanicum* and *R. sanguineus* collected at locality 3**

**Table S7**. Properties of the networks shown in **Figure 3b**: a) Frequency table of clusters in the tick species *H. lusitanicum* network in locality 3. b) Frequency table of clusters in the tick species *R. sanguineus* network in locality 3. c) Detected hub nodes in both groups. d)-f) Centrality values of the bacteria genera with the highest centrality in decreasing order. The tables contain the five bacteria genera with the highest centrality in *H. lusitanicum* and the lower part with the highest centrality in *R. sanguineus* respectively. Thus, a genus can occur twice in the same table (Peschel el at., 2021).

| **a) Cluster at *H. lusitanicum*** | |
| --- | --- |
| **Name** | **Frequency** |
| 0 | 4 |
| 1 | 15 |
| 2 | 25 |
| 3 | 2 |

| **b) Cluster at *R. sanguineus*** | |
| --- | --- |
| **Name** | **Frequency** |
| 1 | 30 |
| 2 | 16 |

| **c) Hub nodes. Based on empirical quantiles of centralities** | |
| --- | --- |
| ***H. lusitanicum*** | ***R. sanguineus*** |
| *Alistipes* | *Akkermansia* |
| *Porphyromonas* | *Bacteroides* |
| *Treponema* | *Campylobacter* |

| **d) Degree** | | |
| --- | --- | --- |
| **Genus** | | |
| **Highest values on the *H. lusitanicum* group:** | ***H. lusitanicum*** | ***R. sanguineus*** |
| *Porphyromonas* | 0.665 | 0.755 |
| *Faecalibacterium* | 0.644 | 0.822 |
| *Alistipes* | 0.622 | 0.688 |
| *Treponema* | 0.577 | 0.711 |
| C. *Udeobacter* | 0.577 | 0.844 |
| **Highest values on the *R. sanguineus* group:** | ***H. lusitanicum*** | ***R. sanguineus*** |
| *Massilia* | 0.555 | 0.866 |
| C. *Udeobacter* | 0.577 | 0.844 |
| *Dialister* | 0.422 | 0.844 |
| *Capnocytophaga* | 0.000 | 0.844 |
| *Arenimonas* | 0.422 | 0.844 |

| **e) Betweenness centrality** | | |
| --- | --- | --- |
| **Genus** | | |
| **Highest values on the *H. lusitanicum* group:** | ***H. lusitanicum*** | ***R. sanguineus*** |
| *Porphyromonas* | 0.058 | 0.103 |
| C. *Xiphinematobacter* | 0.056 | 0.030 |
| *Alistipes* | 0.053 | 0.084 |
| *Faecalibacterium* | 0.053 | 0.120 |
| *Treponema* | 0.045 | 0.016 |
| **Highest values on the *R. sanguineus* group:** | ***H. lusitanicum*** | ***R. sanguineus*** |
| *Dialister* | 0.004 | 0.152 |
| *Faecalibacterium* | 0.054 | 0.120 |
| *Peptostreptococcus* | 0.000 | 0.115 |
| *Porphyromonas* | 0.058 | 0.103 |
| *Capnocytophaga* | 0.000 | 0.093 |

| **d) Eigenvector centrality** | | |
| --- | --- | --- |
| **Genus** | | |
| **Highest values on the *H. lusitanicum* group:** | ***H. lusitanicum*** | ***R. sanguineus*** |
| *Treponema* | 1.000 | 0.869 |
| *Alistipes* | 0.977 | 0.858 |
| *Porphyromonas* | 0.974 | 0.915 |
| *Filifactor* | 0.972 | 0.784 |
| *Faecalibacterium* | 0.958 | 0.827 |
| **Highest values on the *R. sanguineus* group:** | ***H. lusitanicum*** | ***R. sanguineus*** |
| *Akkermansia* | 0.562 | 1.000 |
| *Campylobacter* | 0.700 | 0.951 |
| *Bacteroides* | 0.853 | 0.950 |
| *Streptococcus* | 0.936 | 0.947 |
| *Parabacteroides* | 0.082 | 0.946 |

**Table S8.** Comparison of network properties considering the tick species *H. lusitanicum* and *R. sanguineus* collected from locality 3 (**Figure 3b**) for group differences. The table shows the results from testing global network metrics, their absolute difference, and their respective p-values. A p-value <0.05 indicates species-specific network measures are statistically different. Global network properties are defined for the whole network and offer information on the overall network structure (Peschel et al., 2021).

| **Properties** | ***H. lusitanicum*** | ***R. sanguineus*** | **Absolute difference** | **p-value** |
| --- | --- | --- | --- | --- |
| **Number of components** | 6.000 | 1.000 | 5.000 | 0.059 |
| **Clustering coefficient** | 0.699 | 0.818 | 0.119 | 0.428 |
| **Modularity** | 0.117 | 0.051 | 0.065 | 0.654 |
| **Positive edge percentage** | 64.804 | 56.579 | 8.226 | 0.214 |
| **Edge density** | 0.336 | 0.668 | 0.331 | 0.261 |
| **Natural connectivity** | 0.238 | 0.460 | 0.222 | 0.226 |

**Table S9.** Results from analyses testing for differences between centrality measures of the species-specific networks shown in **Figure 3b**. These results contain the 10 subjects with the highest absolute group difference. All measures are normalized to [0,1]. Table shows the computed measures for *H. lusitanicum* and *R. sanguineus* in locality 3, their absolute difference, and their respective p-values (Peschel et al., 2021). A p-value <0.05 indicates species-specific network measures are statistically different. The p-values are adjusted for multiple testing using the adaptive Benjamini-Hochberg method (Benjamini and Hochberg, 2000), according to Langaas et al. (2005).

|  | ***H. lusitanicum*** | ***R. sanguineus*** | **Absolute difference** | **p-value** |  |
| --- | --- | --- | --- | --- | --- |
| **Degree (normalized)** | | | | | |
| *Capnocytophaga* | 0.000 | 0.844 | 0.844 | 0.074 |  |
| *Parabacteroides* | 0.111 | 0.778 | 0.667 | 0.083 |  |
| *Pyramidobacter* | 0.222 | 0.844 | 0.622 | 0.083 |  |
| *Arenimonas* | 0.244 | 0.844 | 0.600 | 0.074 |  |
| *Peptostreptococcus* | 0.222 | 0.800 | 0.578 | 0.083 |  |
| *Flexilinea* | 0.156 | 0.644 | 0.489 | 0.083 |  |
| *Lactobacillus* | 0.333 | 0.778 | 0.444 | 0.083 |  |
| *Phocaeicola* | 0.022 | 0.467 | 0.444 | 0.083 |  |
| *Lysobacter* | 0.333 | 0.778 | 0.444 | 0.083 |  |
| *Coxiella* | 0.000 | 0.444 | 0.444 | 0.083 |  |
| **Betweenness centrality (normalized)** | | | | | |
| *Dialister* | 0.004 | 0.153 | 0.148 | 1.000 |  |
| *Peptostreptococcus* | 0.000 | 0.115 | 0.115 | 1.000 |  |
| *Capnocytophaga* | 0.000 | 0.094 | 0.094 | 1.000 |  |
| *Leptotrichia* | 0.000 | 0.085 | 0.085 | 1.000 |  |
| *Faecalibacterium* | 0.054 | 0.120 | 0.066 | 1.000 |  |
| *Anaeroglobus* | 0.000 | 0.056 | 0.056 | 1.000 |  |
| *Lysobacter* | 0.009 | 0.061 | 0.051 | 1.000 |  |
| *Fretibacterium* | 0.013 | 0.060 | 0.046 | 1.000 |  |
| *Porphyromonas* | 0.058 | 0.103 | 0.045 | 1.000 |  |
| *Parabacteroides* | 0.000 | 0.040 | 0.040 | 1.000 |  |
| **Closeness centrality (normalized)** | | | | | |
| C. *Xiphinematobacter* | 1.346 | 11.757 | 10.411 | 0.000 |  |
| *Pseudolabrys* | 1.811 | 11.749 | 9.938 | 0.000 |  |
| *Capnocytophaga* | 0.000 | 7.756 | 7.756 | 0.146 |  |
| *Bacteroides* | 2.341 | 7.783 | 5.442 | 0.188 |  |
| *Peptostreptococcus* | 1.400 | 6.833 | 5.433 | 0.175 |  |
| *Lactobacillus* | 1.690 | 6.728 | 5.038 | 0.233 |  |
| *Streptococcus* | 2.709 | 7.340 | 4.630 | 0.233 |  |
| *Porphyromonas* | 2.304 | 6.732 | 4.428 | 0.182 |  |
| *Dialister* | 1.866 | 6.107 | 4.240 | 0.233 |  |
| *Tannerella* | 2.118 | 6.142 | 4.024 | 0.233 |  |

**Table S10.** The Jaccard index quantifies the similarity of the sets of most central nodes and the sets of hub taxa between the microbial networks of tick species *H. lusitanicum* vs. *R. sanguineus* collected in locality 3. Jaccard’s index is 0 if the sets of top-ranking taxa for each centrality measure are completely different and 1 for exactly equal sets. P(J ≥j) indicates the probability of obtaining Jaccard’s index equal to or greater than the observed value under the null hypothesis of no difference between networks ((P(J ≤ j) is deﬁned analogously) (Peschel et al., 2021).

|  | **Jaccard index** | **P(J ≤ Jacc)** | **P(J ≥ Jacc)** |
| --- | --- | --- | --- |
| **Degree** | 0.222 | 0.231 | 0.898 |
| **Betweenness centrality** | 0.278 | 0.412 | 0.768 |
| **Closeness centrality** | 0.333 | 0.608 | 0.587 |
| **Eigenvector centrality** | 0.333 | 0.608 | 0.587 |
| **Hub taxa** | 0.000 | 0.087 | 1.000 |

**Table S11.** The Adjusted Rand index (ARI) quantifies the similarity between clustering structures in the *H. lusitanicum* and *R. sanguineus* microbial networks collected from locality 3, with values close to 1 indicating high agreement or identical clustering and 0 to the expected value for two random clusterings. Consequently, positive values imply that two clusters are more similar, and negative values are less similar than expected at random. The p-value was calculated via permutation (n=1000) to test the significance of the observed clustering agreement (Peschel et al., 2021).

| **Adjusted Rand index** | |
| --- | --- |
|  | Whole network |
| **ARI** | 0.659 |
| **p-value** | 0.000 |

**Additional results for whole network properties of *H. lusitanicum* and *R. sanguineus* collected at locality 4**

**Table S12.** Properties of the networks shown in **Figure 3c:** a) Frequency table of clusters in the tick species *H. lusitanicum* network in locality 4. b) Frequency table of clusters in the tick species *R. sanguineus* network in locality 4. c) Detected hub nodes in both groups. d)-f) Centrality values of the bacteria genera with the highest centrality in decreasing order. The upper part of the table contains the five bacteria genera with the highest centrality in *H. lusitanicum* and the lower part with the highest centrality in *R. sanguineus* respectively. Thus, a genus can occur twice in the same table (Peschet el at., 2020).

| **a) Cluster at *H. lusitanicum*** | |
| --- | --- |
| **Name** | **Frequency** |
| 1 | 25 |
| 2 | 9 |

| **b) Cluster at *R. sanguineus*** | |
| --- | --- |
| **Name** | **Frequency** |
| 1 | 16 |
| 2 | 13 |
| 3 | 5 |

| **c) Hub nodes. Based on empirical quantiles of centralities** | | |
| --- | --- | --- |
| ***H. lusitanicum*** | ***R. sanguineus*** |  |
| *Fusobacterium* | *Clostridium ss 1* |  |
| *Porphyromonas* | *Fusobacterium* |  |

| **d) Degree** | | |
| --- | --- | --- |
| **Genus** | | |
| **Highest values on the *H. lusitanicum* group:** | ***H. lusitanicum*** | ***R. sanguineus*** |
| *Prevotella* | 0.878 | 0.787 |
| *Filifactor* | 0.878 | 0.969 |
| *Udaeobacter* | 0.878 | 0.909 |
| *Fusobacterium* | 0.848 | 1.000 |
| *Fretibacterium* | 0.848 | 1.000 |
| **Highest values on the *R. sanguineus* group:** | ***H. lusitanicum*** | ***R. sanguineus*** |
| *Fusobacterium* | 0.848 | 1.000 |
| *Porphyromonas* | 0.818 | 1.000 |
| *Fretibacterium* | 0.848 | 1.000 |
| *Streptococcus* | 0.757 | 1.000 |
| *Dialister* | 0.818 | 1.000 |

| **e) Betweenness centrality** | | |
| --- | --- | --- |
| **Genus** | | |
| **Highest values on the *H. lusitanicum* group:** | ***H. lusitanicum*** | ***R. sanguineus*** |
| *Bacteroides* | 0.217 | 0.000 |
| *Clostridium* ss *1* | 0.198 | 0.149 |
| *Gaiella* | 0.181 | 0.000 |
| *Fusobacterium* | 0.107 | 0.001 |
| *Lachnospiraceae* NK4A136 | 0.058 | 0.000 |
| **Highest values on the *R. sanguineus* group:** | ***H. lusitanicum*** | ***R. sanguineus*** |
| *Lactobacillus* | 0.000 | 0.157 |
| *Clostridium ss 1* | 0.198 | 0.149 |
| *Porphyromonas* | 0.000 | 0.102 |
| *Treponema* | 0.022 | 0.035 |
| *Filifactor* | 0.013 | 0.034 |

| **f) Eigenvector centrality** | | |
| --- | --- | --- |
| **Genus** | | |
| **Highest values on the *H. lusitanicum* group:** | ***H. lusitanicum*** | ***R. sanguineus*** |
| *Fusobacterium* | 1.000 | 0.945 |
| *Porphyromonas* | 0.942 | 0.941 |
| *Filifactor* | 0.935 | 0.838 |
| *Pyradomibacter* | 0.931 | 0.692 |
| *Prevotella* | 0.931 | 0.796 |
| **Highest values on the *R. sanguineus* group:** | ***H. lusitanicum*** | ***R. sanguineus*** |
| *Clostridium ss 1* | 0.764 | 1.000 |
| *Fusobacterium* | 1.000 | 0.945 |
| *Porphyromonas* | 0.942 | 0.941 |
| *Lactobacillus* | 0.608 | 0.935 |
| *Prevotella* | 0.904 | 0.933 |

**Table S13.** Comparison of network properties considering tick species *H. lusitanicum* and *R. sanguineus* collected from locality 4 (**Figure 3c**) for group differences. The table shows the results from testing global network metrics, their absolute difference, and their respective p-values. A p-value <0.05 indicates species-specific network measures are statistically different. Global network properties are defined for the whole network and offer information on the overall network structure (Peschel et al., 2021).

| **Properties** | ***H. lusitanicum*** | ***R. sanguineus*** | **Absolute difference** | **p-value** |
| --- | --- | --- | --- | --- |
| **Number of components** | 1.000 | 1.000 | 0.000 | 1.000 |
| **Clustering coefficient** | 0.857 | 9.921 | 0.064 | 0.396 |
| **Modularity** | 0.023 | 0.015 | 0.007 | 0.801 |
| **Positive edge percentage** | 65.926 | 73.819 | 7.893 | 0.049 |
| **Edge density** | 0.684 | 0.891 | 0.207 | 0.207 |
| **Natural connectivity** | 0.416 | 0.479 | 0.063 | 0.495 |

**Table S14.** Results from analyses testing for differences between centrality measures of the species-specific networks shown in **Figure 3c**. These results contain the 10 subjects with the highest absolute group difference. All measures are normalized to [0,1]. Table shows the computed measures for *H. lusitanicum* and *R. sanguineus* in locality 4, their absolute difference, and their respective p-values (Peschel et al., 2021). A p-value <0.05 indicates species-specific network measures are statistically different. The p-values are adjusted for multiple testing using the adaptive Benjamini-Hochberg method (Benjamini and Hochberg, 2000), according to Langaas et al. (2005).

|  | ***H. lusitanicum*** | ***R. sanguineus*** | **Absolute difference** | **p-value** |
| --- | --- | --- | --- | --- |
| **Degree (normalized)** | | | | |
| *Peptostreptococcus* | 0.061 | 0.970 | 0.909 | 0.264 |
| *Francisella* | 0.030 | 0.727 | 0.697 | 0.634 |
| *Alistipes* | 0.121 | 0.818 | 0.697 | 0.490 |
| *Johnsonella* | 0.061 | 0.727 | 0.667 | 0.634 |
| *Lachnospiraceae NK4A136 group* | 0.333 | 0.939 | 0.606 | 0.490 |
| *Bacteroides* | 0.333 | 0.848 | 0.515 | 0.490 |
| *Lactobacillus* | 0.667 | 0.970 | 0.303 | 0.490 |
| *Streptococcus* | 0.758 | 1.000 | 0.242 | 0.490 |
| *Clostridium sensu stricto 1* | 0.758 | 0.970 | 0.212 | 0.594 |
| *Coxiella* | 0.818 | 0.606 | 0.212 | 0.674 |
| **Betweenness centrality (normalized)** | | | | |
| *Bacteroides* | 0.218 | 0.000 | 0.218 | 0.104 |
| *Gaiella* | 0.182 | 0.000 | 0.182 | 0.104 |
| *Lactobacillus* | 0.000 | 0.157 | 0.157 | 0.104 |
| *Fusobacterium* | 0.108 | 0.002 | 0.106 | 0.626 |
| *Porphyromonas* | 0.000 | 0.102 | 0.102 | 0.626 |
| *Lachnospiraceae NK4A136 group* | 0.059 | 0.000 | 0.059 | 0.313 |
| *Capnocytophaga* | 0.051 | 0.000 | 0.051 | 0.626 |
| *Clostridium sensu stricto 1* | 0.199 | 0.150 | 0.049 | 0.626 |
| *Peptostreptococcus* | 0.000 | 0.027 | 0.027 | 0.626 |
| *Prevotella* | 0.000 | 0.021 | 0.021 | 0.917 |
| **Closeness centrality (normalized)** | | | | |
| *Clostridium sensu stricto 1* | 1.706 | 3.774 | 2.068 | 0.667 |
| *Lactobacillus* | 1.281 | 3.011 | 1.730 | 0.333 |
| *Fusobacterium* | 2.895 | 1.472 | 1.423 | 0.968 |
| *Porphyromonas* | 2.360 | 3.775 | 1.414 | 0.968 |
| *Tannerella* | 2.421 | 1.437 | 0.983 | 0.968 |
| *Flexilinea* | 1.956 | 1.128 | 0.828 | 0.968 |
| *Fretibacterium* | 2.288 | 1.476 | 0.812 | 0.968 |
| *Anaeroglobus* | 1.914 | 1.177 | 0.737 | 0.968 |
| *Francisella* | 0.000 | 0.665 | 0.665 | 0.968 |
| *Johnsonella* | 0.365 | 0.926 | 0.561 | 0.968 |

**Table S15.** The Jaccard index quantifies the similarity of the sets of most central nodes and the sets of hub taxa between the microbial networks of tick species *H. lusitanicum* vs. *R. sanguineus* collected in locality 4. Jaccard’s index is 0 if the sets of top-ranking taxa for each centrality measure are completely different and 1 for exactly equal sets. P(J ≥j) indicates the probability of obtaining Jaccard’s index equal to or greater than the observed value under the null hypothesis of no difference between networks ((P(J ≤ j) is deﬁned analogously) (Peschel et al., 2021).

| **Properties** | **Jaccard index** | **P(J ≤ j)** | **P(J ≥ j)** |
| --- | --- | --- | --- |
| **Degree** | 0.222 | 0.377 | 0.856 |
| **Betweenness centrality** | 0.286 | 0.475 | 0.738 |
| **Closeness centrality** | 0.385 | 0.758 | 0.447 |
| **Eigenvector centrality** | 0.636 | 0.991 | 0.038 |
| **Hub taxa** | 0.333 | 0.740 | 0.703 |

**Table S16.** The Adjusted Rand index (ARI) quantifies the similarity between clustering structures in the *H. lusitanicum* and *R. sanguineus* microbial networks collected from locality 4, with values close to 1 indicating high agreement or identical clustering and 0 to the expected value for two random clusterings. Consequently, positive values imply that two clusters are more similar, and negative values are less similar than expected at random. The p-value was calculated via permutation (n=1000) to test the significance of the observed clustering agreement (Peschel et al., 2021).

| **Adjusted Rand index** | |
| --- | --- |
|  | **Whole network** |
| **ARI** | 0.220 |
| **p-value** | 0.003 |
